# Astragaloside IV attenuates ferroptosis after subarachnoid hemorrhage *via* Nrf2/HO-1 signaling pathway

**DOI:** 10.3389/fphar.2022.924826

**Published:** 2022-08-19

**Authors:** Zhuanghua Liu, Zhaopeng Zhou, Pu Ai, Chunlei Zhang, Junhui Chen, Yuhai Wang

**Affiliations:** Department of Neurosurgery, The 904th Hospital of PLA, School of Medicine, Anhui Medical University, Wuxi, China

**Keywords:** astragaloside IV, subarachnoid hemorrhage, ferroptosis, early brain injury, nuclear factor erythroid-2 related factor 2

## Abstract

Subarachnoid hemorrhage (SAH) is a severe type of stroke featuring exceptionally high rate of morbidity and mortality due to the lack of effective management. Ferroptosis can be defined as a novel iron-dependent programmed cell death in contrast to classical apoptosis and necrosis. Astragaloside IV (AS-IV) is an active ingredient extracted from Astragalus membranaceus with established therapeutic effect on CNS diseases. However, the exact role of ferroptosis in Astragaloside IV-mediated neuroprotection after SAH is yet to be demonstrated. In the present study, the SAH model of SD male rats with endovascular perforation was used to gauge the neuroprotective effect of AS-IV on SAH-induced early brain injury (EBI) and to clarify the potential molecular mechanism. We found that the induction of SAH reduced the levels of SLC7A11 and glutathione peroxidase 4 (GPX4) in the brain, exacerbated iron accumulation, enhanced lipid reactive oxygen species (ROS) level, and stimulated neuronal ferroptosis. However, the administration of AS-IV and the ferroptosis inhibitor Ferrostatin-1 (Fer-1) enhanced the antioxidant capacity after SAH and suppressed the accumulation of lipid peroxides. Meanwhile, AS-IV triggered Nrf2/HO-1 signaling pathway and alleviated ferroptosis due to the induction of SAH. The Nrf2 inhibitor ML385 blocked the beneficial effects of neuroprotection. These results consistently suggest that ferroptosis is profoundly implicated in facilitating EBI in SAH, and that AS-IV thwarts the process of ferroptosis in SAH by activating Nrf2/HO-1 pathway.

## 1 Introduction

Subarachnoid hemorrhage (SAH) is a severe type of stroke with notoriously high morbidity and mortality rate, thus imposing a heavy burden on society and the patient’s family (Daou et al., 2019; Liu et al., 2022). Early brain injury (EBI) refers to the direct injury in the whole brain that occurs within 72 h after the induction of SAH and is regarded as the major cause of the dismal prognosis of SAH patients (Rass and Helbok, 2019; Qu et al., 2021). Converging evidence suggested a myriad of pathological mechanisms are involved in the pathology of EBI, including apoptosis, autophagy, and pyroptosis (Liu et al., 2020; Wang et al., 2021). Although efforts have been made to develop many drugs targeting these mechanisms, the prognosis of patients suffering from SAH has not been improved considerably (Daou et al., 2019; Neifert et al., 2021). In such circumstances, it is necessary to apply and initiate other feasible strategies to improve the neurological function of patients after SAH.

Ferroptosis is a novel form of programmed cell death caused by dysregulated control of membrane LPO (lipid peroxidation). Ferroptosis is characterized by an iron-dependent and oxidative form of regulated cell death (RCD), mostly triggered by accumulated lipid peroxidation and membrane damage in eukaryotic organisms (Dixon et al., 2012; Lu et al., 2021). Morphologically speaking, ferroptosis is manifested by cell membrane rupture and blistering, mitochondrial membrane density compression, mitochondrial cristae atrophy or even disappearance, and mitochondrial outer membrane rupture (Lu et al., 2021; Liu et al., 2022). Its biochemical features mainly include dysfunction of iron ion metabolism, glutathione (GSH) depletion, iron-dependent accumulation of lipid reactive oxygen species (ROS), and inhibited or decreased glutathione peroxidase 4 (GPX4) activity (Chen et al., 2020; Zhang Y. et al., 2021). Recent studies have confirmed that ferroptosis can frequently occur in the central nervous system (CNS), and is thus implicated in a set of CNS disorders, including aging, tumor development and stroke (Chen et al., 2020; Tan et al., 2021). Several recent investigations have shown that iron deposition and lipid peroxide accumulation tend to increase 24 h after SAH, and that the ferroptosis inhibitor Ferrostatin-1 (Fer-1) upregulates the levels of SLC7A11 and GPX4 to rescue SAH-induced neuronal damage (Kuang et al., 2021; Li et al., 2021). Therefore, the development of drugs that target the process of ferroptosis is a promising direction for the clinical intervention of EBI.

**FIGURE 1:**
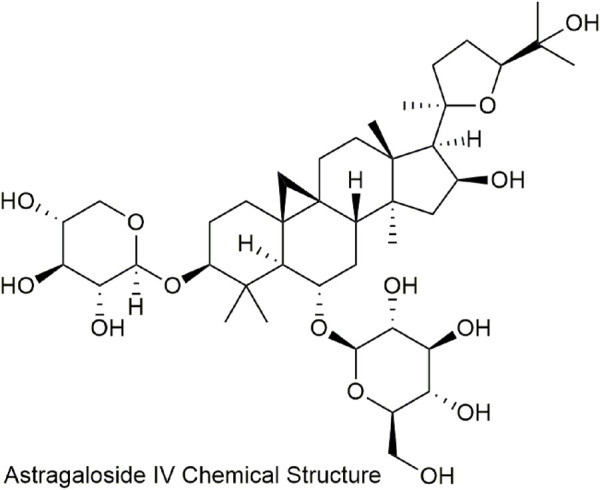
The chemical structure of Astragaloside IV.

Many natural compounds have been reported to exhibit therapeutic functions by modulating ferroptosis (Zhang S. et al., 2021; Ge et al., 2021; He et al., 2021). Astragaloside IV (AS-IV), as a newly found glycoside of cycloartane-type triterpene, is the effective component extracted from *Astragalus membranaceus* (Costa et al., 2019) (Figure 1). The therapeutic function of AS-IV has been demonstrated in several neuron degenerative disorders (such as Alzheimer’s Disease, Parkinson’s Disease, Cerebral Ischemia) and Autoimmune Encephalomyelitis (Costa et al., 2019). Zhu et al. (2008) indicated that the potent suppressive effect of AS-IV on spontaneous neuronal excitabilities is likely to underlie the potential mechanism of its neuroprotective effect. Intriguingly, emerging evidence indicated that rats with SAH showed significant improvement with regard to learning, memory, motor status, and neurological function after 24 h of AS-IV treatment (Yang et al., 2020). Moreover, the treatment of AS-IV could effectively alleviate brain edema after SAH at 24 h. These findings concur that AS-IV plays a potential neuroprotective role against SAH.

Nrf2 pathway contributes to the neuroprotective function against oxidative stress in a variety of CNS diseases, including SAH (Zolnourian et al., 2019). Keap1-Nrf2 pathway is known as the regulator of cyto-protective response to ROS-induced endogenous and exogenous stresses (Dodson et al., 2019). Under non-stress condition, Kelch-like ECH-associated protein 1 (Keap1) binds to Nrf2 in the cytoplasm to stimulate the degradation of Nrf2 via ubiquitin proteasome pathway. Once stimulated, however, Nrf2 will dissociate from the sequestering of Keap1 to enter the nucleus, where it binds to the antioxidant response element (ARE), and activate a set of cytoprotective enzymes to protect cells from oxidative damage (Chen et al., 2020). Accumulating evidence suggests that the liberation of Nrf2 from Keap1, its cytoplasmic repressor will provoke Nrf2 accumulation in the nucleus (Zolnourian et al., 2019; Jiang et al., 2020).

In addition, Nrf2 is also involved in other vital signaling pathways, including lipid metabolism, energy metabolism and iron homeostasis, all of which are precisely localized to regulate ferroptosis (Lu et al., 2021). Liu et al. (2021) found that α-Lipoic Acid functions to ameliorate the process of ferroptosis in the MPP^+^-induced PC12 cells by elevating PI3K/Akt/Nrf2 signaling. Yuan et al. (2021) demonstrated that the activated Nrf2/SLC7A11/GPX4 pathway is ascribed to alleviating Oxygen-Glucose Deprivation/Reoxygenation (OGD/R)- induced Neuronal Ferroptosis. A number of studies have confirmed that AS-IV plays a protective role against adriamycin-induced myocardial fibrosis by thwarting the process of ferroptosis (Luo et al., 2021). Notably, the neuroprotective effect of AS-IV treatment after SAH could be attributable, at least in part, to the activation of Nrf2/HO-1 signaling pathway. However, whether AS-IV counteracts ferroptosis after SAH by mediating Nrf2/HO-1 pathway remains obscure. In the present study, we intend to validate the plausible neuroprotective mechanism of AS-IV inhibiting ferroptosis after SAH by activating the Nrf2/HO-1 pathway.

## 2 Material and methods

### 2.1 Animals

Adult male SD rats (10–12 weeks, 250–300 g) were raised and fed *ad libitum* at a constant temperature of 23–25 °C in the animal house with a 12-h light/ dark cycle. All the rats were granted free access to water and food. The experiments and protocols of the study have been approved by the Ethics Committee of Wuxi Clinical College of Anhui Medical University. All procedures were performed in accordance with animal welfare guidelines of NIH for the handling and care of animals in the lab. Effort has been made to minimize the suffering of the rats.

### 2.2 Subarachnoid hemorrhage model

**FIGURE 2:**
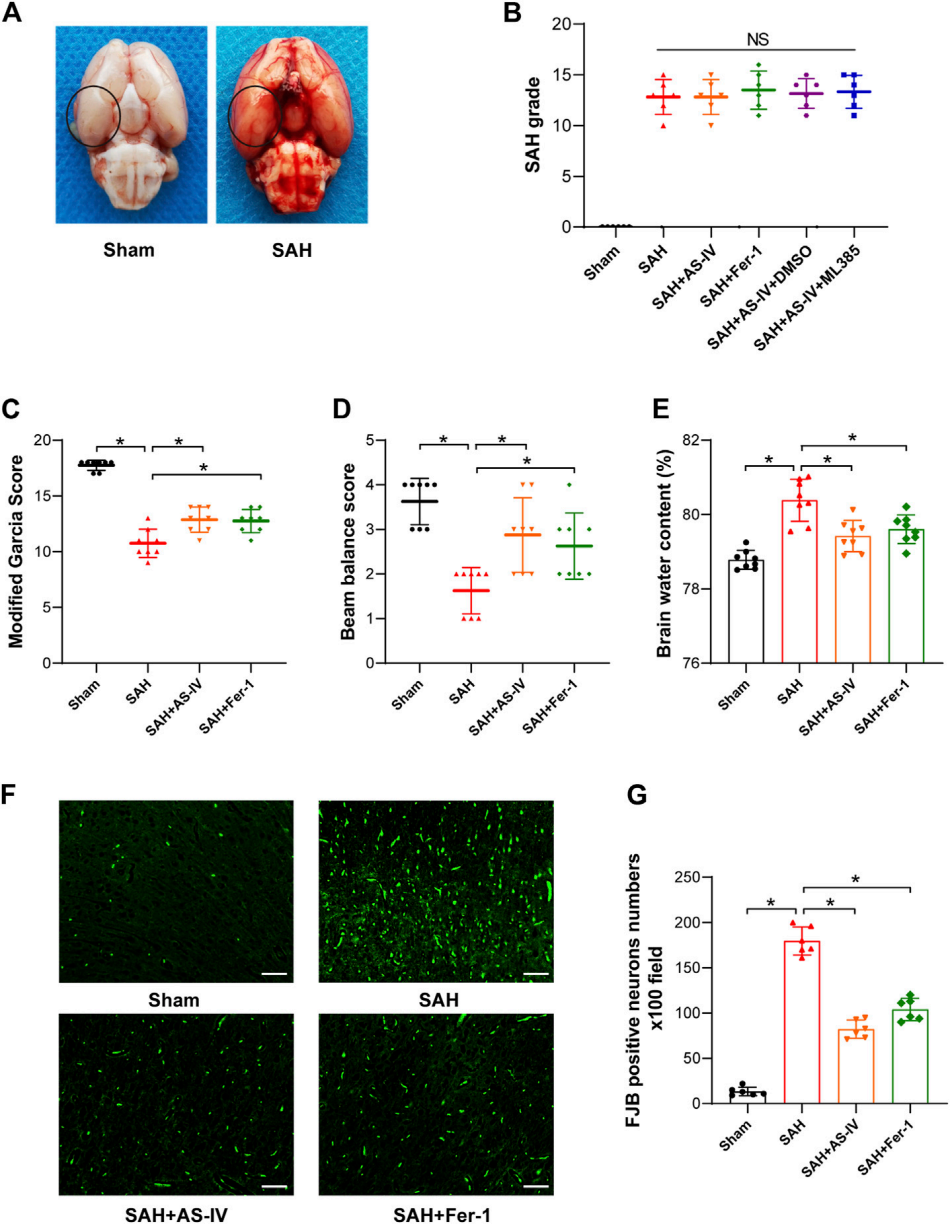
AS-IV and Fer-1 treatment improve EBI after SAH in rats. **(A)** Representative brain tissue images of rats in the Sham and SAH groups, the area in the circle represents the sampling site for Western blot and kit assay. **(B)** SAH grade scores 24 h after induction of SAH in rats (*n* = 6). **(C,D)** Quantitative measurement of modified Garcia score and beam balance score (*n* = 8). **(E)** Quantification of brain water content in various groups of rats at 24h post-SAH (*n* = 8). **(F)** Representative images of FJB staining in the right temporal cerebral cortex of rats (scale bar = 50 μm). **(G)** Quantitative measurement of FJB staining (*n* = 6). *p < 0.05; FJB, Fluoro-Jade B**.**

SAH model was constructed by applying endovascular perforation in the rats, according to the protocol introduced in a previous study (Wei et al., 2020), except for slight modifications. Briefly, after performing intraperitoneal anesthesia with 40 mg/kg sodium pentobarbital, the right common carotid, external and internal carotid arteries of the rats were exposed and isolated. The right external carotid artery was ligated, and a 4–0 single-strand nylon thread was used to insert the right internal carotid artery through the stump of the external carotid artery and the bifurcation of the common carotid artery. When resistance is felt when the suture enters the intracranial segment, proceed approximately 3 mm to penetrate internal carotid artery at the bifurcation of middle cerebral artery. The suture was held in this position for 10 s and was then withdrawn. The rats in the Sham group went through an identical procedure, without the suture at the point of resistance. Throughout the experiment, the body temperature of the rats was sustained at around 37 °C by using a thermal blanket. After the wounds were sutured, the rats were placed in a separate cage and neurological function was closely observed. Rats were euthanized 24 h after SAH surgery to obtain cortical brain tissue for subsequent experiments (Figure 2A).

### 2.3 Experimental design

In Experiment no. 1, 88 rats (a total of 112 underwent surgery and 24 were excluded due to death or failure to meet the SAH grade threshold) were randomly and evenly subdivided into the following four groups: Sham group (n = 22), SAH group (n = 22), SAH + AS-IV group (n = 22), and SAH + Fer-1 group (n = 22). At 24 h after modeling, 8 rats in each group were scored for neurological function and then for the detection of brain water content; 8 rats in each group were used for Western blot assay and the detection of iron, ROS, GSH and MDA; the remaining in each group were used to detect Fluoro-Jade B (FJB) and immunofluorescence (IF).

In experiment no. 2, 110 rats (a total of 141 underwent surgery and 31 were excluded due to death or failure to reach the SAH grade threshold) were randomly and evenly assigned into the following five groups: sham group (n = 22), SAH group (n = 22), SAH + AS-IV group (n = 22), SAH + AS-IV + DMSO group (n = 22), and SAH + AS-IV + ML385 group (n = 22). Similarly, all rats were euthanized 24 h after successful modeling, 8 rats in each group were tested for neurological function scores, and then they were used for the detection of brain edema; 6 rats in each group were used for FJB and IF staining, and the remaining 8 rats were used for Western blot and related kit detection.

### 2.4 Drug administration

All doses and methods of administration were based on the protocols of previous experiments (Zhang et al., 2019b; Zhang et al., 2019c; Kuang et al., 2021). AS-IV (No. 84687–43-4, purity ≥98%) was obtained from Nanjing spring and autumn biotech Co. Ltd. (Nanjing, China). The injection of AS-IV (20 mg/kg) was carried out *intraperitoneally* (i.p.) at 30 min after the induction of SAH. Fer-1 was purchased from Selleckchem (Houston, TX, United States). Fer-1 (dosage = 2 mg/kg) were administrated i.p. After 30 min of operation. The rats in the sham group were administrated with an equal amount of normal saline (NS). ML385 (50 pmol/5 μl; AOBIOUS, Gloucester, MA, United States), a specific Nrf2 inhibitor, was diluted in DMSO, and was used for intracerebroventricular (i.c.v.) injection at 24 h before SAH.

### 2.5 Grading system for Subarachnoid hemorrhage

According to the previous study (Liu et al., 2020), SAH grades were assessed by an independent investigator, who was blind to the grouping of the animals. Briefly, the basal part of the rats’ brain could be subdivided into 6 regions, each of which was scored according to the degree of subarachnoid hemorrhage: 0, no subarachnoid hemorrhage; 1, a small amount of subarachnoid hemorrhage; 2, moderate hemorrhage with notable arteries; 3, a blood clot that covers all the arteries in the segment. The scores were calculated as the summation of these six-zone scores, with the grade ranging from 0 to 18. where rats with SAH classification scores below 8 were excluded from the study.

### 2.6 Neurological score

Based on a previous study (Liu et al., 2020), we used the modified Garcia score to evaluate the neurological function of rats 24 h after the induction of SAH. The modified Garcia’s scoring system mainly covers the following six indicators: 1) symmetry of limb movement; 2) forelimb extension activity; 3) spontaneous activity; 4) climbing ability; 5) trunk touch response, and 6) whisker touch response. The score for each indicator ranges from 0 to 3. Hence the maximum value is 18. In addition, a beam balance test was used to assess the exercise capacity of the rats by measuring the distance that the rats walked on the wood within 1 min. The score ranges from 0 to 4, and a higher score indicates the superior athletic ability.

### 2.7 Brain water content

The severity of cerebral edema was commonly assessed by using the dry/ wet weight method. Immediately after perfusion, the whole brain was removed. Each brain tissue was quickly weighed, and the procedure was triplicated to obtain the average value as the wet weight. We then place the brain tissue in a drying oven for a 24-h dehydration at 105°C. The tissue was weighed to obtain the dry weight. The brain water content of rats could reasonably be calculated according to the formula below: brain water content = [(wet weight-dry weight)/ (wet weight)] ×100%.

### 2.8 Immunofluorescence staining

Under deep anesthesia, the rats were perfused with paraformaldehyde and the brains were harvested. Brain tissues were fixed in 4% paraformaldehyde for 24 h and embedded in paraffin. Coronal sections (4 μm) were treated with xylene and gradient alcohol, then blocked with 5% bovine serum albumin for 45 min at room temperature. The sections were incubated with the following primary antibodies, including anti-GPX4 (1:200, cat.no. A1933, Abclonal; anti-NeuN (1:200, cat.no. MAB377, Merck Millipore); anti-Nrf2 (1:100, cat.no. A11159, Abclonal) overnight at 4°C. Subsequently, after being rinsed three times with PBST, the sections were dried, and the fluorescent secondary antibody was added dropwise for incubation at 37°C for 1 h in a wet box, then 4,6-diamidino-2-phenylindole (DAPI) was added for incubation at room temperature for 15 min. Finally, we sealed the slides using a mounting liquid that contains the anti-fluorescence quencher. The right temporal cerebral cortex images were observed, photographed and collected under the fluorescence microscope.

### 2.9 FJB staining

FJB staining is commonly used to detect Neuronal death by identifying degenerating neurons in the right temporal cerebral cortex (Kuang et al., 2021). First, the brain tissue sections were placed on slides, and then the sections were soaked in different concentrations of ethanol for rehydration. After being washed by using distilled water for 2 min, the sections were treated with 0.06% potassium permanganate solution for 10 min. The sections were repeatedly rinsed using distilled water for prolonged 2 min, and were placed in 0.0004% FJB solution (Sigma-Aldrich, United States) for incubation. The tissue sections were subsequently rinsed three times with distilled water, allowed to dry and then sealed with neutral resins. Sections were examined with a fluorescent microscope, and images were captured. The counting and statistics of FJB (+) neurons were performed by a researcher blinded to the experimental groups.

### 2.10 Detection of iron concentration

Abiding by the product manufacturer’s instructions, we used an iron detection kit (TC1015, Leagene Biotechnology Co. Ltd. China) to detect iron content in the tissues. Briefly, after homogenizing and loading the appropriate tissue, the absorbance of the collected sample was measured at the wavelength of 562 nm, and the concentration of iron was calculated accordingly.

### 2.11 Lipid ROS measurement

ROS levels in brain tissue were detected using a ROS kit (Beyotime, China). Briefly, brain tissue samples were homogenized and centrifuged at 10,000 g, 4°C for 15 min. ROS levels in tissues were detected using DCFH-DA according to the manufacturer’s instructions. Fluorescence intensity was detected using a fluorescent microplate reader (Molecular Devices, United States) with excitation wavelength of 485 nm and emission wavelength of 530 nm. All data were normalized to the Sham group.

### 2.12 Contents of malondialdehyde (MDA) and GSH measurements

Levels of MDA and GSH in brain tissue were investigated by using commercialized Assay kits (Beyotime, China) 24 h after SAH, in accordance with the manufacturer’s instructions. The absorbance of MDA in the samples was detected at 532 nm, and the concentration of GSH was determined at 412 nm, respectively.

### 2.13 Nuclear and cytoplasmic protein Extractions

According to the manufacturer’s instructions, nuclear and cytoplasmic proteins were extracted using the Nuclear and Cytoplasmic Protein Extraction Kit (Beyotime, China). Briefly, fresh brain tissue was first thoroughly homogenized with tissue homogenate (60 mg tissue/200 μl) and placed in an ice bath for 15 min. After centrifugation at 1,500g for 5 min at 4°C, the supernatant was extracted to obtain a fraction of the cytoplasmic protein. Then, the precipitate was homogenized with a cytoplasmic protein extraction reagent, centrifuged (4°C, 15,000 g, 5 min) and the obtained supernatant was the remaining cytoplasmic protein. Total cytoplasmic protein was the sum of the two. Finally, the pellet was suspended using a nuclear protein extraction reagent, centrifuged (4°C, 15,000 g, 10 min), and the resulting supernatant was nuclear protein.

### 2.14 Western blot assay

Western blot was performed according to the standard protocol described in a previous study (Xie et al., 2019). The collected ipsilateral cerebral cortical samples were fully lysed using protease inhibitors (Beyotime, China) and RIPA lysis buffer (Thermo Fisher, United States). An equal amount of protein was separated in each lane by performing SDS-PAGE, and the protein was transferred onto PVDF membrane (Millipore, United States). The membrane was blocked by using 5% nonfat milk for 1 h at RT, and then was incubated with primary antibody overnight at 4°C. The membrane was washed with TBST for three times, each of which lasted 5 min. Afterwards, secondary antibody was added for 2 h of incubation at RT. The intensity of signal was detected by chemiluminescence using ECL substrate (Vazyme, China). ImageJ software was used for the comparison of signal intensity. The following primary antibodies were used: anti-SLC7A11 (1:500, cat#A15604; Abclonal); anti-GPX4, (1:500, cat# A1933, Abclonal); Anti-Nrf2 (1:1000, cat# A11159, Abclonal); Anti-Histone H3 (H3), (1:5000, cat# AF0009, Beyotime); Anti-HO-1 (1:1000, cat# ab13248, Abcam); Anti-Tubulin, (1:2000, cat# 2148S, Cell signaling technologies); Anti-GAPDH (1:5000, cat# 5174S, Cell signaling technologies).

### 2.15 Statistical analysis

GraphPad Prism 8.0 was used for statistical analysis in the current study. All the data are presented in the form of mean ± standard deviation (SD). After testing the data for normality, the comparison between the two groups was carried out by using Student’s t-test. One-way ANOVA was used for comparisons between multiple groups, followed by Tukey post hoc analysis. *p* value of less than 0.05 was considered to indicate a significant difference.

## 3 Results

### 3.1 Mortality and subarachnoid hemorrhage grade

253 rats in total were enrolled in the experiment, of which 41 deceased in less than 24 h after induction of SAH, and 14 with a SAH grade of less than 8 were excluded to ensure rigor and consistence. As shown in Table 1, none of the rats in the Sham group died, and the overall mortality rate of rats with SAH was 19.62% (41/209). SAH grades were not significantly disparate among the 5 groups of SAH (Figure 2B).

**TABLE 1:** Animal usage and mortality.

Groups	Mortality	Excluded

Experiment 1		
Sham	0(0/22)	0
SAH	22.58%(7/31)	2
SAH + ASIV	16.67%(5/30)	3
SAH + Fer-1	20.69%(6/29)	1

Experiment 2		
Sham	0(0/22)	0
SAH	20%(6/30)	2
SAH + ASIV	17.24%(5/29)	2
SAH + ASIV + DMSO	17.86%(5/28)	1
SAH + ASIV + ML385	21.88%(7/32)	3

Total		
Sham	0(0/44)	0
SAH	19.62%(41/209)	14

### 3.2 astragaloside IV treatment improves early brain damage after subarachnoid hemorrhage

After the induction of SAH, the modified Garcia scoring and beam balance test scores of the rats were significantly reduced, indicating that SAH significantly impaired the neurological function and motor ability of rats. Conversely, AS-IV treatment significantly improved neurological scores after SAH (Figures 2C,D). Moreover, brain water content was applied to indicate the severity of brain edema. The results showed that rats developed significant brain edema after SAH. However, AS-IV could significantly alleviate brain edema after SAH (Figure 2E). FJB staining showed that 24 h after SAH, neuronal death in the cerebral cortex was significantly increased, while AS-IV treatment could significantly alleviate neuronal death after SAH (Figures 2F,G). To explore whether AS-IV was associated with ferroptosis, we used the ferroptosis inhibitor Fer-1 as a positive control. Interestingly, Fer-1 treatment exerted a similar effect as AS-IV (Figures 2C–G).

### 3.3 astragaloside IV treatment alleviates ferroptosis after subarachnoid hemorrhage

**FIGURE 3:**
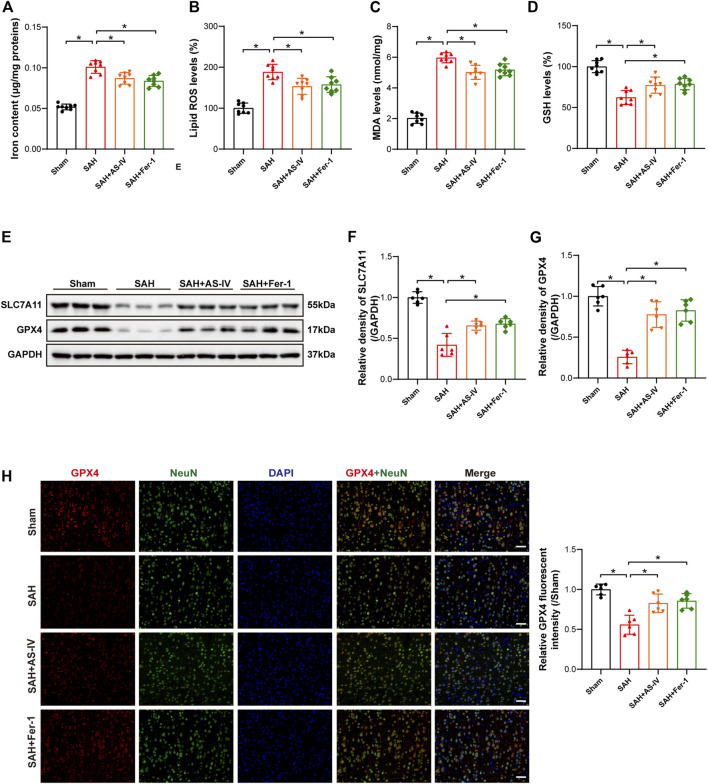
AS-IV and Fer-1 treatment attenuated ferroptosis 24 h after SAH. **(A)** Quantitative analysis of iron concentrations after SAH (n = 8). **(B–D)** Quantitative analysis of lipid ROS, MDA, and GSH levels (n = 8). **(E–G)** Western blot analysis of protein expression levels of SLC7A11 and GPX4 (n = 6). **(H)** Typical double immunofluorescence images of GPX4 and NeuN (scale bar = 50 μm). Data are represented as mean ± SD. *p < 0.05, NS: not statistically significant.

The results of iron concentration detection showed that the iron content of brain tissue was significantly increased after SAH, while AS-IV and ferroptosis inhibitor Fer-1 could significantly reduce its content (Figure 3A). The levels of Lipid ROS and MDA were significantly increased in the SAH group, as opposed to the Sham group. However, AS-IV and Fer-1 treatment could significantly down-regulate their levels (Figures 3B,C). In addition, the rats in the SAH group showed significantly reduced concentration of GSH in the brains. However, the levels of GSH in AS-IV and Fer-1 treated rats were considerably increased than that in SAH rats (Figure 3D). Therefore, AS-IV treatment appears to reduce iron content and alleviate lipid peroxidation in rat brains after SAH.

Western blot results revealed that the protein expressions of SLC7A11 and GPX4 were significantly reduced in SAH rats, and AS-IV and Fer-1 treatment could significantly increase the expression levels of SLC7A11 and GPX4 (Figures 3E–G). Double immunofluorescence staining confirmed our Western blot findings. Meanwhile, GPX4 was colocalized with the neuronal marker NeuN, indicating that GPX4 was expressed in neurons (Figure 3H).

### 3.4 The administration of astragaloside IV activated Nrf2/ HO-1 signaling pathway

**FIGURE 4:**
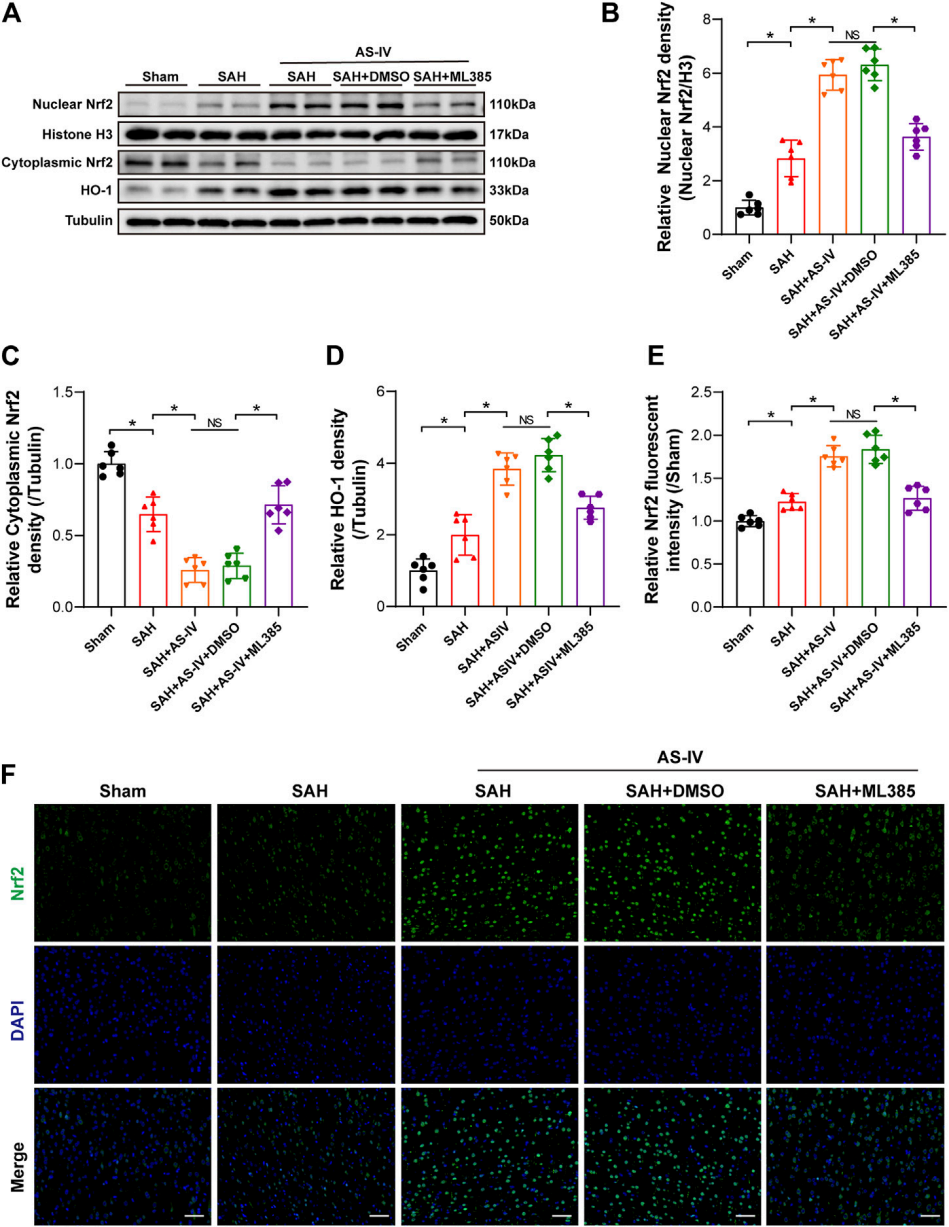
AS-IV treatment enhances Nrf2/HO-1 signaling pathway at 24 h post-SAH in rats. **(A)** Representative Western blotting images of Nuclear Nrf2, Cytoplasmic Nrf2, and HO-1. **(B–D)** Quantification of Nuclear Nrf2, Cytoplasmic Nrf2, and HO-1 (n = 6). **(E)** Quantification of Nrf2 immunofluorescence staining (n = 6). **(F)** Representative immunofluorescence images of Nrf2 (scale bar = 50 μm). *p < 0.05, NS: not statistically significant; DMSO: dimethyl sulfoxide.

To explore the molecular mechanism whereby AS-IV alleviates SAH, we examined the key members in Nrf2/ HO-1 Signaling pathway. It was found that SAH significantly elevated the expression of Nuclear Nrf2 and HO-1, whereas suppressed the expression of Cytoplasmic Nrf2, in comparison with the Sham group. The expression levels of Nuclear Nrf2 and HO-1 were increased to a greater extent after AS-IV treatment (Figures 4A,B,D). Consistently, the level of Cytoplasmic Nrf2 protein in SAH + AS-IV group was significantly down-regulated than SAH group (Figures 4A,C). ASIV treatment promotes the intranuclear transfer of Nrf2 in senescent cells, yet, ML385, an inhibitor of Nrf2, diminishes the activation of Nrf2/ HO-1 signaling pathway induced by the administration of AS-IV (Figures 4A–D). Immunofluorescence staining further confirmed that Nrf2 was mostly expressed in the cytoplasm in the Sham group, yet ASIV treatment potently facilitated Nrf2 translocation into the nucleus (Figures 4E,F).

### 3.5 ML385 diminishes the protective effect of astragaloside IV on early brain injury after induction of subarachnoid hemorrhage

**FIGURE 5:**
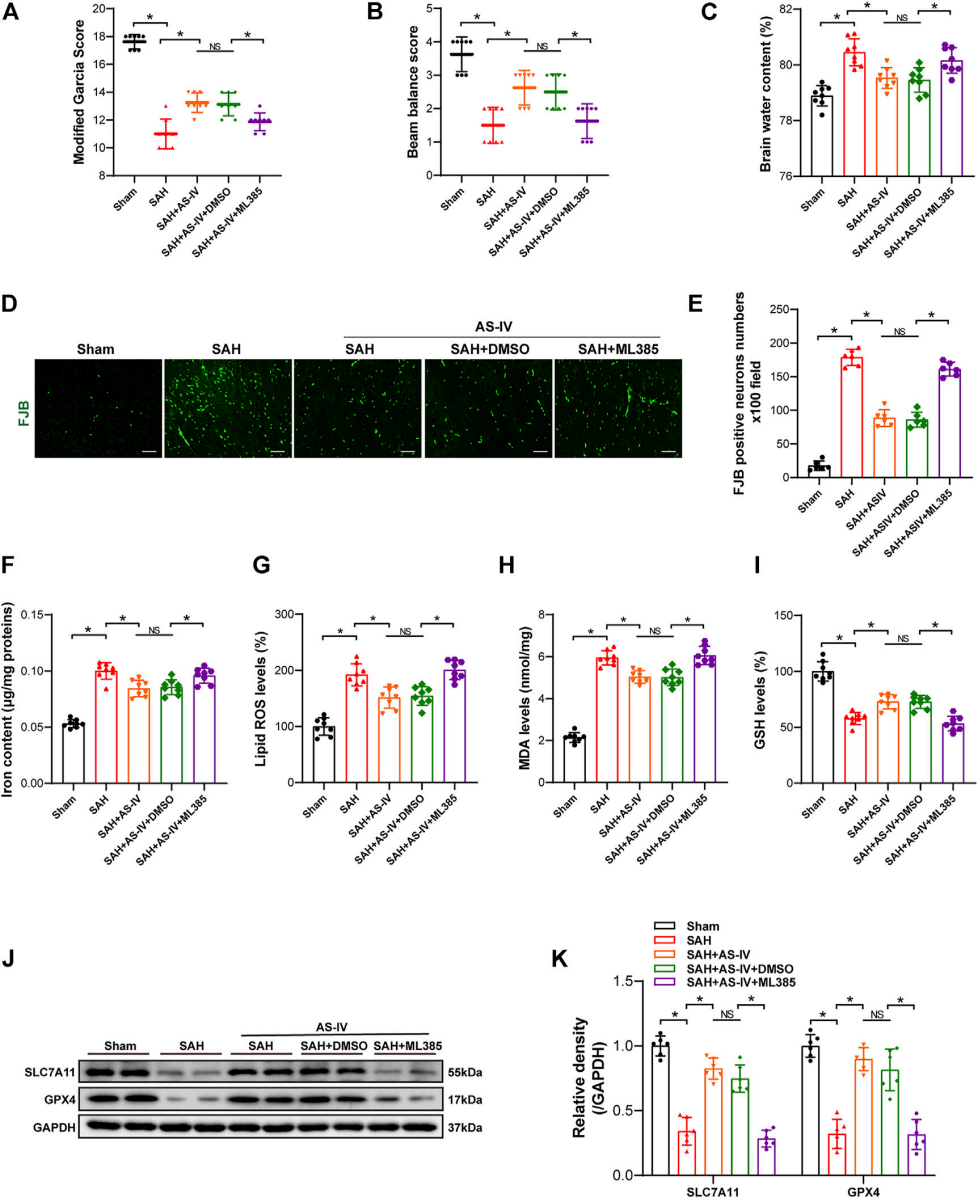
ML385 inhibits the neuroprotective effect of AS-IV at 24 h post-SAH. **(A–C)** Modified Garcia scores, beam balance scores, and brain water content 24 h after SAH in rats (*n* = 8). **(D,E)** Representative images and quantitative analysis of FJB staining in right temporal cerebral cortex of rats (n = 6, scale bar = 50 μm). **(F)** Quantification of iron concentrations in rats after SAH (*n* = 8). **(G–I)** Quantitative analysis of lipid ROS, MDA, and GSH levels (*n* = 8). **(J)** Representative Western blot images of SLC7A11 and GPX4. **(K)** Quantitative analysis of SLC7A11 and GPX4 (*n* = 6). Data are represented as mean ± SD. *p < 0.05, NS: not statistically significant; DMSO: dimethyl sulfoxide; FJB, Fluoro-Jade B**.**

Intriguingly, the modified Garcia score and beam balance test scores were significantly lower in the SAH + AS-IV + ML385 group than in SAH + AS-IV group. Such a trend indicates that ML385 could diminish the neuroprotective effect produced by AS-IV (Figures 5A,B). Meanwhile, the brain water content of rats in the SAH + AS-IV + ML385 group was significantly increased than that in the SAH + AS-IV group (Figure 5C). FJB staining showed that Nrf2 inhibitor led to significantly aggravated neuronal death (Figures 5D,E). These data reveal that AS-IV could alleviate EBI through activating Nrf2/ HO-1 Signaling pathway, whereas ML385 diminished the protective effect of AS-IV on EBI induced by SAH.

### 3.6 ML385 diminishes the protective effect of astragaloside IV that attenuates ferroptosis after subarachnoid hemorrhage

Iron content, Lipid ROS and MDA levels in SAH + AS-IV + ML385 group were significantly higher than SAH + AS-IV group (Figures 5F–H). Conversely, the GSH level in SAH + AS-IV + ML385 group was significantly lower, as opposed to that in SAH + AS-IV group (Figure 5I). Western blot showed that the inhibitor that targets Nrf2/ HO-1 signaling pathway significantly down-regulated the expression of SLC7A11 and GPX4 proteins (Figures 5J,K). Thus, we found that ML385 counteracted the protective effect of AS-IV that thwarts ferroptosis after SAH.

## 4 Discussion

In the current study, we validate the neuroprotective effect of AS-IV on early-stage SAH, which is evidenced by improvements in neurological function, motor ability, as well as reduced brain edema and less neuronal death in SAH model of rats. Meanwhile, AS-IV and the ferroptosis inhibitor Fer-1 could enhance the antioxidant capacity after SAH and inhibit the accumulation of iron content and lipid peroxides, thereby improving ferroptosis. Of note, our data showed that AS-IV activated Nrf2/ HO-1 signaling pathway, yet the Nrf2/ HO-1 pathway inhibitor ML385 could neutralize the protective effect of AS-IV via resisting ferroptosis after SAH. Therefore, the present study provides concrete evidence to shed light on the therapeutic effect of AS-IV to lessen the severity of SAH-induced EBI by attenuating ferroptosis and activating Nrf2/ HO-1 pathway.

The intricate molecular mechanism involved in the etiology and pathology of EBI after induction of SAH continues to defy the understanding of researchers. In recent years, a large number of studies have been performed to study EBI from the perspective of alleviating neuronal apoptosis, neuroinflammation, autophagy, and pyroptosis, yet these methods have not achieved satisfactory long-term prognosis in patients suffering from SAH (Zolnourian et al., 2019; Wei et al., 2020; Wang et al., 2021). Therefore, more efforts are needed to gain insight into the molecular mechanism of EBI after SAH, thereby devising potential strategies for clinical intervention. In 2012, Dixon et al. (2012) reported, for the first time, a novel form of iron-dependent programmed cell death (known as ferroptosis), which is deeply implicated in the occurrence and development of CNS diseases. This finding provides insights into the management of EBI after SAH. Zille et al. (2017) reported that experimental intracerebral hemorrhage (ICH) has the characteristics of ferroptotic and necroptotic cell death, without depending on apoptosis or autophagy; and that ferroptotic and necroptotic inhibitors could contribute to cell survival. A large number of recent studies confirmed that ferroptosis is involved in the onset and progression of SAH-induced EBI and that the administration of ferroptosis inhibitors, Fer-1 and Liproxstatin-1, exhibit potent protective effect against EBI (Cao et al., 2021; Kuang et al., 2021; Li et al., 2021). In this study, we found that AS-IV, along with the ferroptosis inhibitor Fer-1, could significantly improve neurological impairment, exercise capacity, brain edema, neuronal death, and EBI in rats with SAH.

Ferroptosis is mostly caused by a disturbance of iron homeostasis and accumulation of Lipid ROS in the cytoplasm (Dixon et al., 2012; Ge et al., 2021). The massive hemorrhage causes brain tissue compression and promotes neuronal damage. Throughout the process, the excessive release of heme and hemoglobin (Hb) from lysed red blood cells has been observed to react with HO-1 to produce Fe^2+^. When the content of iron exceeds storage capacity, labile iron pool (LIP) level will increase, subsequently aggravating neuronal damage and death via promoting the secretion of ROS (Qiu et al., 2020; Hu et al., 2021). GPX4, a selenium-containing enzyme and an integral reducer of lipid peroxides, is known to play a vital part in reducing the production of lipid ROS. Intriguingly, GPX4, by consuming two GSH molecules, interacts with the toxic LOOHs (lipid hydroperoxides), converting into the harmless L-OHs (non-toxic lipid alcohols) (Zhang Y. et al., 2021; Tan et al., 2021). The activity of GPX4 is the cornerstone of antiperoxidant defence, given that the inhibition of GPX4, directly or indirectly, tends to induce ferroptosis. Moreover, the hindered synthesis of GSH, a prevailing antioxidant and a cofactor of GPX4, will lead to GSH depletion and ultimately ferroptosis. The constitutive amino acids that participate in the synthesis of GSH include, glycine (Gly), cysteine (Cys), and glutamate (Glu). Among these three amino acids, cysteine (Cys) is considered to be the key factor that restrains the level of GSH. Specifically, extracellular Cys is transported into the cell through cystine/glutamate antiporter (System Xc-) on the cell membrane, whereas intracellular Glu is exported out of the cell (Tan et al., 2021; Liu et al., 2022). In the event of thwarted transportation, GSH could be depleted, thereby rendering GPX4 unable to yield the harmless LOHs via reduction of the toxic LOOHs. Badgley et al. reported that deletion of SLC7A11, a subunit of System Xc^−^, could induce ferroptosis and suppress the progression of pancreatic ductal adenocarcinoma (Badgley et al., 2020). Our study showed that AS-IV treatment significantly reduced iron content, Lipid ROS, and MDA levels after SAH, and ameliorated ferroptosis by bolstering the levels of GSH, GPX4 and SLC7A11.

Subsequently, we explored the possible molecular mechanism that underlies the function of AS-IV in the treatment of SAH. Transcription factor Nrf2 is a classic protective regulator acting to maintain cellular redox homeostasis by mediating the expression of a series of anti-oxidant enzymes and detoxification genes. In the presence of oxidative stress, Nrf2 will be translocated from the cytoplasm to the nucleus, thereby participating in the transcription of a series of ARE-related genes, which are involved in the regulation of iron metabolism, glutathione synthesis, and lipid peroxidation (Ge et al., 2021; Lu et al., 2021). The activation of Nrf2 pathway reduces intracellular iron pool to restore iron homeostasis, limits ROS production, and upregulates SLC7A11 (Dodson et al., 2019; Zhang S. et al., 2021). HO-1 is one of the downstream target genes of Nrf2 with cytoprotective and antioxidant properties (Parfenova et al., 2012). Jiang et al. (2020) demonstrated that gastrodin could protect HT-22 neurons from the damage of glutamate-induced ferroptosis via activating Nrf2/HO-1 signaling pathway. Yan et al. (2021) found that dimethyl fumarate alleviated inflammation, oxidative stress, and ferroptosis through the Nrf2/ARE/NF-κB pathway, thereby alleviating cognitive impairment in rats with chronic cerebral hypoperfusion. Several recent studies have shown that Nrf2 is expressed in neurons (Zhang et al., 2019a; Zhang Z. H. et al., 2021). However, the mechanism by which the Nrf2/ HO-1 signaling pathway is involved in the neuronal ferroptosis after induction of SAH remains uncertain. Our study found that AS-IV promoted the translocation of Nrf2 from cytoplasm to the nucleus and resulted in elevated levels of HO-1. Such finding suggests that the therapeutic function of AS-IV is manifested through activating Nrf2/HO-1 signaling pathway.

To validate that AS-IV protected against the EBI after SAH by inhibiting ferroptosis through the Nrf2/HO-1 signaling pathway, we applied the Nrf2-specific inhibitor ML385 to block the pathway. The results showed that AS-IV treatment improved neurological function and motor capacity, reduced brain edema and neuronal death, and the protective effects were diminished by ML385. In addition, AS-IV treatment reduced iron contents and lipid peroxide levels after SAH and increased the levels of GSH, GPX4 and SLC7A11 to inhibit ferroptosis. However, ML385 diminished the inhibitory effect of AS-IV on ferroptosis. The results indicated that Nrf2/HO-1 signaling pathway was profoundly implicated in exhibiting the neuroprotective effect of AS-IV on EBI after SAH by partially regulating ferroptosis. However, the molecular mechanism by which AS-IV exerts neuroprotective effects on SAH warrants more intensive analysis in the future.

**FIGURE 6:**
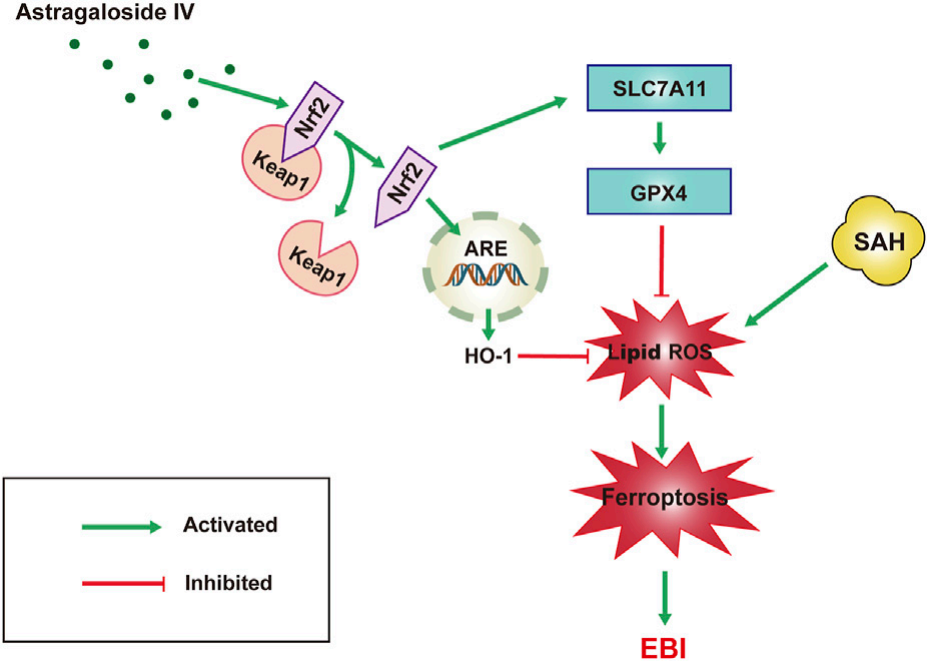
Schematic representation of the neuroprotective effect of AS-IV treatment after SAH. AS-IV inhibits ferroptosis via enhancing Nrf2/HO-1 signaling pathway, thereby ameliorating EBI 24 h after SAH in rats.

Our results suggest that the neuro-protective effect of AS-IV to assuage EBI induced by SAH is most probably attributable to the anti-ferroptotic action *via* triggering Nrf2/HO-1 pathway (Figure 6). This study helps to ascertain that the inhibition of ferroptosis could be the molecular mechanism justifying the protective effects of AS-IV against 444 EBI induced by SAH.

## Data Availability

The original contributions presented in the study are included in the article/Supplementary Material, further inquiries can be directed to the corresponding authors.
