# Supplementary material for: Astragaloside IV attenuates ferroptosis after subarachnoid hemorrhage via Nrf2/HO-1 signaling pathway
Source: Front Pharmacol. 2022 Aug 19;13:924826. doi: 10.3389/fphar.2022.924826 (PMC9437486; doi:10.3389/fphar.2022.924826)
Supplement: Supplementary file 1 [file DataSheet2.docx]

The following are links to Supplemental files:

https://www.jianguoyun.com/p/DT3Ym9MQ0IbAChiFoswEIAA
